# Reframing Obesity as Collective Vulnerability in Black Churches in The USA: Implications for Clergy Leadership, Health Equity, and Pastoral Identity

**DOI:** 10.1007/s10943-026-02673-8

**Published:** 2026-04-28

**Authors:** Linda Carper, Naa-Solo Tettey, Harold Griffin

**Affiliations:** 1 Public Health Department, Walden University, 100 Washington Ave S, Suite 1210, Minneapolis, MN 55401 USA; 2 Department of Public Health, William Paterson University, 300 Pompton Road, Wayne, NJ 07470 USA

**Keywords:** Syndemic theory, Black church, Clergy leadership, Obesity disparities, Theological interpretation

## Abstract

The COVID-19 pandemic exposed and intensified longstanding racial health inequities in the USA, disproportionately affecting African American communities with high burdens of chronic disease. This study examines how African American clergy in Houston, Texas, interpreted and responded to the convergence of COVID-19 and obesity-related vulnerability within their congregations, and how these conditions reshaped theological meaning, pastoral identity, and health-related leadership within Black church contexts. Using a hermeneutic interpretive phenomenological approach, in-depth interviews were conducted with ten Protestant clergy engaged in health-related ministry during the pandemic. Findings indicated that clergy experienced COVID-19 not only as a public health crisis but as a theological disruption that reshaped pastoral priorities. Four interrelated patterns emerged: Obesity was reframed from a lifestyle issue to a matter of survival and collective vulnerability; clergy became more aware of embodied vulnerability; health engagement accelerated in response to congregational loss; and clergy expanded their role as trusted interpreters of public health information amid medical mistrust. Chronic disease prevention shifted from a secondary activity to a core dimension of pastoral identity. Although behavioral concepts such as decision-making and confidence were evident, they were shaped by theological meaning, moral responsibility, and commitment to the congregation. These findings suggest that crisis conditions can transform how health is understood and addressed within Black churches. Efforts to reduce chronic disease disparities and advance health equity should engage faith institutions not only as partners in outreach but as central contributors to how health meaning is formed within communities shaped by structural inequity.

## Introduction

The COVID-19 pandemic did not generate health inequities in the USA; it exposed and intensified them. Patterns of infection, hospitalization, and mortality followed longstanding lines of racialized structural inequality. African American communities experienced disproportionate morbidity and mortality shaped by occupational exposure, residential segregation, unequal access to preventive care, and elevated burdens of chronic disease (Bailey et al., 2019; Nardone et al., 2020). Conditions such as obesity, hypertension, type 2 diabetes, and cardiovascular disease were already prevalent prior to 2020 and emerged as powerful predictors of severe illness and death during the pandemic (Tremmel et al., 2017; Trust for America’s Health, 2019). COVID-19 operated not simply as an infectious outbreak but as a syndemic event in which biological vulnerability and structural inequality converged to amplify harm (Gravlee, 2020; Horton, 2020; Singer et al., 2017).

Syndemic theory emphasizes that epidemics do not unfold in isolation. They interact with preexisting patterns of inequality and chronic disease to produce concentrated risk within marginalized populations (Singer et al., 2017; Tsai, 2018). For many African American communities, the pandemic made visible how structural racism becomes biologically embodied over time (Krieger, 2011). Obesity and related metabolic conditions were not isolated clinical variables but markers of cumulative exposure to constrained food environments, chronic stress, environmental inequity, and limited healthcare access (Tettey et al., 2017). When COVID-19 emerged, these underlying vulnerabilities became immediately consequential.

### The Black Church and Pastoral Authority in Crisis

Within this context, Black churches served as critical sites of interpretation, mobilization, and response. Historically, these institutions have functioned as more than places of worship, operating as parallel civic structures that provide political leadership, social services, and collective identity in the face of exclusion (American Public Health Association, 2022; Berkley-Patton et al., 2022; Lincoln & Mamiya, 1990). During periods of crisis, congregations often serve as stabilizing infrastructures through which communities process uncertainty and coordinate action (Billingsley, 1999).

Clergy leadership within these institutions carries a form of relational authority that differs from biomedical expertise. Pastoral credibility is cultivated through long-term presence across life events, including illness, bereavement, and economic hardship. In communities shaped by justified medical mistrust rooted in historical exploitation (Gamble, 1997; Hammond et al., 2021), pastors often serve as interpretive bridges between public health guidance and congregational meaning systems (Tettey et al., 2017). Their influence rests on relational trust and theological fluency rather than clinical credentialing.

Prior to the pandemic, chronic disease prevention within many congregations was often positioned as an auxiliary ministry. Health fairs, walking programs, and educational workshops existed; yet, discussions of obesity and metabolic illness competed with other spiritual and social priorities (Tettey et al., 2016). Weight-related disparities were acknowledged but not consistently framed as existential threats to congregational continuity. Obesity occupied a complex space that was visible, culturally mediated, and often entangled with stigma, requiring pastoral sensitivity to identity, dignity, and communal tradition. The pandemic shifted this balance.

### Shifting Meaning, Leadership, and Theological Response

COVID-19 brought chronic disease into immediate pastoral focus within congregational life. Clergy conducted funerals under distancing restrictions, witnessed disproportionate hospitalization among congregants with metabolic conditions, and responded to questions from families navigating uncertainty. Obesity and hypertension were no longer abstract comorbidities but visible amplifiers of vulnerability in real time. This shift aligns with scholarship on embodied inequality, which shows how social disadvantage becomes physiologically embedded across the life course (Krieger, 2011; Williams & Mohammed, 2013). For many clergy, the pandemic transformed longstanding structural inequities into an immediate moral confrontation.

Many participants described this period as a theological turning point. Scriptural metaphors of the body as temple and vessel took on renewed urgency. Stewardship was interpreted not only as spiritual discipline but as care for embodied life. Sermons incorporated discussions of immune vulnerability and chronic disease risk. Prayer was paired with practical guidance about vaccination and comorbidities, and health literacy became part of pastoral counseling. What had once been peripheral became central to ministry identity.

From a behavioral perspective, the crisis appeared to accelerate processes described within the transtheoretical model. Clergy who had previously considered deeper health engagement described movement toward action following personal loss or congregational illness. Awareness emerged through lived experience rather than formal instruction. Decisional balance shifted as addressing metabolic health became tied to protecting congregational life. At the same time, these shifts were not purely behavioral. They were shaped by theological and relational meaning. Religious coping scholarship suggests that crisis can prompt reinterpretation of suffering and strengthen communal cohesion (Pargament, 2011; Park, 2013), a dynamic reflected in clergy narratives.

Clergy also assumed expanded communicative roles. As public health guidance evolved and misinformation circulated widely, pastors were called upon to interpret epidemiological trends, address skepticism about vaccination, and guide congregational decision making. Churches hosted testing and vaccination events, and safety protocols were communicated through sermons, livestreams, and text messaging networks (Holt et al., 2022; Thomas et al., 2021). In these contexts, pastoral leadership intersected directly with public health practice.

Conversations about obesity also shifted in tone. Rather than centering individual blame, many clergy framed metabolic health in terms of communal protection and ministry longevity. Weight-related risk was discussed in relation to safeguarding elders, sustaining leadership, and preserving congregational continuity. The language of shame receded, replaced by a focus on survival and collective responsibility. This shift altered how weight was situated within theological discourse.

Despite a substantial body of research on faith-based health promotion, most studies have focused on program implementation and intervention outcomes. Less attention has been given to how clergy reinterpret their roles, theological commitments, and leadership priorities under conditions of acute public health crisis. In particular, limited work has examined how syndemic conditions reshape not only health behaviors within congregations but also the meaning systems through which those behaviors are understood and acted upon.

This study addresses that gap by examining how African American clergy in Houston interpreted and responded to the convergence of COVID-19 and chronic disease vulnerability. It integrates syndemic theory, theological interpretation, and behavioral theory to examine how structural inequality, meaning-making, and behavioral change processes intersect within pastoral identity. Rather than treating these frameworks as separate components, this study analyzes how they operate together under crisis conditions to shape theological meaning, leadership, and health-related engagement within Black churches.

By centering clergy narratives, this research positions obesity not simply as a behavioral concern but as a focal point of theological reflection and communal vulnerability. It examines how crisis reorganized perceptions of urgency, authority, and responsibility within Black churches, and how these shifts reshaped pastoral identity and health-related leadership. Understanding this process is essential for developing culturally grounded and spiritually integrated approaches to reducing health disparities in historically marginalized communities.

## Background

### Syndemic Exposure and the Embodiment of Structural Inequity

The disproportionate impact of COVID-19 on African American communities must be understood within a broader ecology of structural inequality and chronic disease accumulation. Longstanding racialized inequities in housing, employment, environmental exposure, food systems, wealth distribution, and health care access have shaped patterns of metabolic vulnerability across generations (Bailey et al., 2019; Nardone et al., 2020). Obesity, hypertension, and type 2 diabetes are not randomly distributed biomedical conditions. They are socially patterned outcomes embedded in geography, policy history, and economic marginalization (Suglia et al., 2016).

Syndemic theory provides a critical framework for understanding how COVID-19 unfolded within this context. Rather than conceptualizing the pandemic as a discrete infectious event, it emphasizes the interaction of co-occurring epidemics within conditions of structural inequality, producing mutually reinforcing harms (Gravlee, 2020; Singer et al., 2017). In African American communities, chronic metabolic illness was already entrenched prior to 2020. The arrival of SARS-CoV-2 intensified these vulnerabilities, revealing the embodied consequences of racial stratification (Krieger, 2021; Williams & Mohammed, 2013).

Within this framework, obesity functions not merely as an individual behavioral concern but as a biological index of accumulated disadvantage. Chronic stress exposure, neighborhood disinvestment, constrained food environments, limited preventive care, and economic instability contribute to sustained metabolic strain. COVID-19 exposed the fragility of bodies shaped by these cumulative conditions. Clergy witnessing disproportionate mortality among congregants were not observing isolated tragedies; they were confronting the material effects of structural inequity in real time.

The pandemic thus operated as both an epidemiological event and a structural revelation. It compressed decades of chronic disease disparity into an acute moral and communal crisis.

### The Black Church as Moral Infrastructure in Crisis

The Black church has historically functioned as moral infrastructure during periods of social disruption. Across slavery, Reconstruction, Jim Crow, the Civil Rights Movement, and contemporary political instability, churches have served as sites of organizing, education, mutual aid, and collective survival (Lincoln & Mamiya, 1990). Their authority is relational rather than bureaucratic. It is cultivated through continuity of presence, pastoral care, and communal embeddedness.

Public health scholarship increasingly recognizes the strategic importance of faith institutions in addressing racial health disparities (American Public Health Association, 2022; Berkley-Patton et al., 2022). Faith-based initiatives have demonstrated measurable impact in domains including cancer screening, hypertension control, HIV prevention, and vaccination uptake (Campbell et al., 2023; Levin, 2023). Importantly, clergy authority operates through trust, an asset of particular significance in communities shaped by justified medical mistrust rooted in historical exploitation and ongoing discrimination (Gamble, 1997; Hammond et al., 2021).

During COVID-19, this historic crisis-response function intensified. Churches rapidly transitioned to digital worship, organized food distribution networks, coordinated mutual aid, and disseminated evolving public health guidance. Clergy became intermediaries between local health systems and congregational life, translating epidemiological data into culturally resonant and theologically coherent messaging (Holt et al., 2022; Thomas et al., 2021). This mediation was not peripheral. It was foundational to collective compliance and protection.

Yet prior to 2020, chronic disease prevention within many congregations remained structurally secondary. Health ministries were often active, but obesity and metabolic illness were not consistently framed as urgent theological imperatives. Chronic disease disparities were acknowledged; yet, they did not uniformly occupy central pastoral concern. The pandemic altered that prioritization.

### Theological Anthropology, Embodiment, and Crisis Reinterpretation

The COVID-19 pandemic provoked renewed theological reflection on embodiment. Within many Black churches, the metaphor of the body as “the temple” has long circulated in preaching and pastoral discourse. Prior to the pandemic, this metaphor frequently operated symbolically rather than programmatically. COVID-19 rendered embodiment concrete. Mortality statistics were no longer abstract. Hospitalizations and funerals reoriented theological reflection toward biological vulnerability.

Practical theological scholarship emphasizes that embodiment is not peripheral to faith but central to lived spirituality (Doehring, 2015; Swinton, 2011). Illness disrupts abstraction. It demands interpretive response. Religious coping theory further suggests that crisis can catalyze meaning reconstruction, moral recalibration, and renewed communal commitment (Pargament, 2011; Park, 2013).

In the context of COVID-19, clergy narratives reflected this reconstructive process. Stewardship expanded beyond moral exhortation toward bodily preservation. It became linked to communal survival. Chronic disease was reframed not as personal shortcoming but as shared vulnerability. Theological discourse shifted from symbolic reference to embodied urgency.

This interpretive shift represents a critical intersection of syndemic exposure and theological anthropology. Structural vulnerability became visible through congregational loss. Theology responded by rearticulating responsibility.

### Crisis as Behavioral Accelerator: Reinterpreting Stage Theory in Faith Contexts

The transtheoretical model (TTM) offers a behavioral framework for understanding how individuals and institutions progress from contemplation to action (Brick et al., 2016). Under typical circumstances, stage progression unfolds incrementally through consciousness raising, environmental reevaluation, and strengthened self-efficacy. COVID-19 disrupted this gradualism.

For many clergy, awareness of chronic disease disparities preceded the pandemic (Tettey et al., 2016). What changed was urgency. Personal loss sharpened decisional balance. The costs of inaction became immediate and visible. Funerals functioned as experiential accelerants. Movement from contemplation to preparation and action was compressed.

Importantly, this progression was not solely cognitive. It was mediated through theological meaning-making. Self-efficacy was framed not merely as individual mastery but as faith-informed obligation. Decisions to initiate health programming, address obesity explicitly, or host vaccination clinics were described as matters of pastoral accountability and obedience rather than lifestyle preference.

This theological embedding complicates secular applications of stage theory. In faith settings, agency is relationally constructed and morally interpreted. Decisional balance incorporates spiritual responsibility. Processes of change unfold within communal identity rather than individual autonomy (Bandura, 1997; Romain et al., 2018). Crisis therefore functioned as both structural pressure and theological catalyst.

### Mistrust, Mediation, and Clergy as Health Translators

Medical mistrust in African American communities is historically grounded and empirically documented (Gamble, 1997; Hammond et al., 2021). During COVID-19, this mistrust intersected with widespread misinformation circulating across digital platforms. Clergy frequently occupied a mediating position, addressing concerns about masking, vaccination, and governmental guidance.

Research demonstrates that trusted community leaders significantly influence health behavior adoption when institutional trust is fragile (Bogart et al., 2021; Quinn et al., 2019). In many congregations, pastors became interpreters of epidemiological uncertainty. Their influence derived not from scientific credentialing but from relational credibility cultivated over decades.

As links between obesity and COVID-19 severity became more widely recognized, clergy reframed metabolic health within broader narratives of preparedness and resilience. Obesity shifted from stigmatized behavioral category to marker of collective vulnerability. The language of protection replaced the language of blame.

### Crisis-Induced Transformation of Pastoral Identity

The pandemic also reorganized pastoral identity. Prior to 2020, health engagement often existed alongside other ministry domains. During COVID-19, it became central. Clergy expanded involvement in health education, institutional partnership building, and public advocacy.

Disaster and emergency scholarship documents how role boundaries expand under crisis pressure (Aten et al., 2019; Gaillard & Texier, 2010). In Black churches, where pastoral authority is embedded in communal survival, this expansion carried theological weight. Health leadership was no longer auxiliary outreach. It was interpreted as shepherding.

This raises a critical empirical and theoretical question: Does crisis-induced mobilization produce sustained transformation, or does it recede as immediacy fades? Existing literature has examined faith-based health promotion and clergy influence on discrete interventions. However, limited scholarship has explored how acute syndemic conditions reorganize theological interpretation, decisional balance, and institutional prioritization within Black churches.

The present study addresses this gap by examining how African American clergy interpreted and responded to the convergence of COVID-19 and obesity-related vulnerability within their congregations. Specifically, this study explores how crisis conditions reshaped theological meaning, pastoral identity, and health-related leadership within Black church contexts.

## Methods

### Methodological Orientation

This study employed a hermeneutic interpretive phenomenological methodology (Creswell & Poth, 2018) to examine how African American clergy made sense of obesity, chronic disease vulnerability, and pastoral responsibility during the COVID-19 pandemic. The purpose was not to evaluate intervention efficacy but to explore how crisis conditions reshaped meaning, identity, and leadership within congregational contexts.

The approach aligns with Heideggerian hermeneutic phenomenology and contemporary interpretive phenomenological analysis (IPA), which assume that experience is always already interpreted and situated (Alase, 2017). Meaning is not accessed in raw form but is mediated through theology, culture, vocation, history, and embodiment. Clergy responses to obesity and pandemic vulnerability are therefore shaped by doctrinal commitments, congregational expectations, racialized health inequities, and lived pastoral practice. A hermeneutic phenomenological orientation allowed for careful attention to how participants interpreted these intersecting dimensions rather than reducing their narratives to behavioral categories or discrete health outcomes.

Although the transtheoretical model informed later analytic reflection, it did not structure the study design or data collection in a prescriptive manner. Behavioral constructs such as decisional balance, self-efficacy, and processes of change were engaged only after inductive themes were established. This sequencing preserved analytic openness to emergent theological and relational meanings while permitting theoretically informed interpretation during later stages of analysis.

### Context and Setting

The study was conducted in Houston, Texas, a large metropolitan region characterized by racial diversity, a dense religious infrastructure, and documented disparities in obesity, cardiovascular disease, and diabetes. Historically Black congregations occupy prominent social and cultural roles within the region, functioning as sites of spiritual formation, political engagement, and community care.

During the COVID-19 pandemic, Houston experienced marked racial disparities in infection, hospitalization, and mortality. This context rendered the city a salient site for examining how syndemic conditions intersected with pastoral leadership and congregational health realities.

### Participant Recruitment and Sampling

Participants were recruited using purposive and network-based sampling through established clergy associations and interfaith coalitions. Recruitment focused on African American clergy serving predominantly African American congregations in Houston, Texas, USA, who were actively engaged in health-related ministry, including efforts related to obesity, chronic disease, or COVID-19 response.

Eligibility criteria included self-identification as African American or Black, a minimum of 10 years of pastoral leadership experience, active service within a predominantly African American congregation, and direct involvement in health-related ministry initiatives. A total of ten clergy met these criteria and participated in the study (Table 1).

**Table 1:** Participant demographic characteristics (*N* = 10)

Characteristic	*n*	%
Race/Ethnicity		
African American / Black	10	100
Gender		
Male	8	80
Female	2	20
Clergy role		
Senior Pastor	7	70
Associate/Assistant Clergy	3	30
Denominational affiliation		
Protestant (multiple denominations)	10	100
Congregation Context		
Predominantly African American congregations	10	100
Geographic location		
Houston, Texas, United States	10	100
Years in Ministry		
≥ 10 years (eligibility criterion)	10	100
Health Ministry Involvement		
Active engagement in health-related ministry	10	100

Participants were African American clergy serving predominantly African American congregations in Houston, Texas, United States (*N* = 10). All participants identified as African American or Black, had ≥10 years of pastoral leadership experience, and were actively engaged in health-related ministry (e.g., obesity, chronic disease, or COVID-19 response efforts). Participants represented multiple Protestant denominations

All participants identified as African American or Black (100%) and served predominantly African American congregations (100%) within a single metropolitan region. The sample included eight male (80%) and two female (20%) clergy. Participants represented multiple Protestant denominations (100%) and varied in ministerial role, including seven senior pastors (70%) and three associate or assistant clergy (30%), allowing for variation in institutional authority and leadership experience. All participants had a minimum of 10 years of pastoral leadership (100%) and were actively engaged in health-related ministry (100%), consistent with eligibility criteria.

In hermeneutic phenomenological research, adequacy is determined by depth of experiential insight and interpretive richness rather than statistical representation (Creswell & Poth, 2018). Data collection and analysis occurred concurrently, and recruitment concluded when additional interviews yielded thematic redundancy and conceptual consistency across participant accounts. Given the small, purposive sample drawn from a single geographic region and across multiple denominational contexts, the findings are intended to provide context-specific, exploratory insight rather than statistical generalizability.

### Researcher Positionality and Reflexivity

Given the interpretive nature of the inquiry, reflexivity was treated as an epistemological commitment rather than procedural addendum. The researcher brings professional experience in public health and faith-based health programming, including prior engagement with clergy-led interventions. This positionality provided contextual fluency and facilitated rapport, yet also required vigilance to avoid theoretical imposition or over-identification.

A reflexive journal was maintained throughout recruitment, interviewing, coding, and thematic development. Entries documented analytic decisions, theological assumptions, emotional responses to narratives of illness and mortality, and potential points of interpretive bias. Reflexive insights were integrated into analytic memos and reviewed during theme refinement to enhance confirmability and analytic transparency.

All coding and analysis were conducted by the primary researcher. Peer debriefing sessions with qualitative research colleagues provided external scrutiny of thematic coherence and theoretical integration, strengthening analytic rigor without fragmenting interpretive consistency.

### Data Collection

Data were collected through in-depth, semi-structured interviews conducted via secure video conferencing to accommodate clergy schedules and public health restrictions. Interviews ranged from approximately 50–75 min and were audio-recorded with participant consent.

The interview guide prioritized narrative elicitation over structured response. Core domains included perceptions of obesity and chronic disease within congregational life; experiences of health leadership prior to and during the pandemic; theological interpretations of the body, stewardship, and vulnerability; personal experiences of illness, fatigue, or loss; observations of congregational resistance or readiness; and shifts in messaging and ministry priorities following pandemic-related mortality.

Probes encouraged reflection on moments of tension, disruption, or change. Rather than asking directly about behavioral stages, questions invited participants to narrate evolving interpretations and decisions across time.

Interviews were transcribed verbatim and reviewed against recordings for accuracy. Identifying information was removed prior to analysis.

Institutional Review Board approval was obtained prior to recruitment. Participants provided informed consent electronically and were reminded of their right to withdraw at any time.

### Analytic Strategy

Analysis followed an iterative, multi-phase process consistent with hermeneutic phenomenological inquiry and thematic analysis (Braun & Clarke, 2006), as well as the double hermeneutic characteristic of interpretive phenomenological analysis.

Each transcript was read in full multiple times to apprehend narrative flow, emotional inflection, theological language, and moments of rupture. Particular attention was given to turning points, including personal diagnoses, congregational loss, and pandemic-related mortality, which appeared to catalyze interpretive shifts.

Transcripts were coded inductively using close, line-by-line analysis. Early codes remained proximate to participant language to preserve phenomenological texture. Examples included embodied witness, ministerial exhaustion, food as fellowship, loss as awakening, and public health translator.

Codes were clustered through constant comparison across cases. Convergences and divergences were examined to identify patterns in how clergy framed vulnerability, authority, responsibility, and embodiment. Analytic memos documented evolving interpretations and supported refinement of thematic domains.

Only after core thematic structures were established were behavioral constructs from the transtheoretical model explicitly engaged. Decisional balance was examined as moral discernment; self-efficacy was reconsidered in relation to theological agency; and processes of change were analyzed within the context of crisis acceleration and relational accountability.

This sequencing ensured that theory illuminated rather than constrained participant meaning. Behavioral constructs were treated as interpretive resources rather than classificatory frameworks.

NVivo qualitative analysis software was used to organize coding, facilitate cross-case comparison, and maintain a structured audit trail documenting analytic evolution.

### Strategies to Enhance Rigor

Credibility was strengthened through prolonged engagement with transcripts, iterative analysis, and member affirmation. Participants were invited to review summarized thematic interpretations; feedback confirmed resonance with lived experience and did not materially alter thematic structure.

Dependability was supported through systematic documentation of coding decisions, reflexive entries, and analytic memos within NVivo. Peer debriefing provided structured challenge to emerging interpretations.

Confirmability was enhanced by maintaining a transparent audit trail linking raw transcript excerpts to thematic claims. Direct quotations were retained to anchor interpretation in participant language.

Transferability was addressed through thick contextual description of setting, congregational dynamics, clergy experience, and regional health disparities, enabling readers to assess applicability to other Black faith communities.

### Ethical Considerations

All transcripts were de-identified prior to analysis. Digital files were stored on password-protected devices in accordance with institutional data security standards. Pseudonyms were assigned, and potentially identifying congregational details were removed from dissemination materials.

Given the emotional salience of narratives involving illness, dialysis, hospitalization, and mortality, interviews were conducted with attentiveness to participant well-being. Clergy was reminded that they could decline to answer any question or pause the interview at any time.

## Results

Analysis of participant narratives examined how African American clergy interpreted and responded to the convergence of COVID-19 and chronic disease vulnerability within their congregations. Clergy described this experience not simply as a public health emergency, but as a theological disruption that reshaped how obesity, embodiment, and pastoral responsibility were understood. Across interviews, participants did not frame weight-related leadership primarily as programmatic or strategic. Instead, they described a process of reinterpretation, moral reckoning, and expanded pastoral responsibility that unfolded in response to visible loss.

Four interrelated domains characterized this shift: (1) from lifestyle issue to existential threat, (2) embodied witness and ministerial exposure, (3) crisis as moral compression, and (4) translational authority under conditions of mistrust (Table 2).

**Table 2:** Thematic findings: interpretive domains of clergy experience

Theme	Description	Illustrative focus	Interpretive Meaning
From Lifestyle Issue to Existential Threat	Obesity reframed from a behavioral concern to a matter of survival and collective vulnerability	Shift from discipline to protection; urgency tied to COVID-19 mortality	Repositions obesity as a collective survival issue rather than an individual behavioral concern
Embodied Witness and Ministerial Exposure	Clergy recognized their own physical vulnerability and the visibility of their health behaviors	Pastoral body as example; lived experience shaping credibility	Links personal embodiment to moral authority, reinforcing the role of lived experience in leadership
Crisis as Moral Compression	Pandemic accelerated awareness and action related to chronic disease	Loss and funerals as catalysts; reduced hesitation to address obesity	Condenses temporal and moral urgency, accelerating shifts in health prioritization and action
Translational Authority Under Conditions of Mistrust	Clergy acted as interpreters of public health information within contexts of medical mistrust	Framing science through theology; trusted communication channels	Positions clergy as mediators who translate institutional knowledge into culturally and theologically meaningful forms
Cross-Domain Integration	Themes operated simultaneously to reshape pastoral identity and responsibility	Obesity reframed within theology, leadership, and communal accountability	Reflects the integration of health, theology, and leadership into a unified framework of pastoral identity

Themes were developed through hermeneutic interpretive phenomenological analysis and reflect interconnected, rather than sequential, processes

These domains did not function as linear stages. Rather, they represent overlapping interpretive movements that collectively reshaped how clergy understood the body, vulnerability, and leadership during the pandemic.

### From Lifestyle Issue to Existential Threat

Across nearly all interviews, obesity was initially described as a recognized but background concern prior to 2020. Clergy acknowledged its prevalence within their congregations, yet typically situated it within broader conversations about diet, discipline, or fellowship culture. The tone was measured and familiar.

Participants consistently described a disruption in this framing once COVID-19 mortality patterns became visible. Chronic disease ceased to function as an abstract risk category and instead became concretely associated with hospitalization and death. Obesity and related metabolic conditions were described as amplifiers of vulnerability.

One pastor reflected, “We realized this wasn’t just about a virus. It was about everything we had been ignoring for years.” Another stated, “When you start seeing who ends up in ICU, you can’t unsee it.”

Participants described this reframing as changing how they understood the body and vulnerability within their theological framework. The body, which had often been referenced metaphorically in sermons, was now seen as more fragile and materially exposed. Many participants spoke about a renewed emphasis on stewardship language, but with a stronger sense of urgency. The idea of the body as a temple was no longer used simply as a rhetorical reminder. Instead, it became closely connected to questions of survival and responsibility during the pandemic.

Several clergy acknowledged that conversations about weight had previously felt delicate or potentially shaming. During the pandemic, that hesitation diminished. Health was reframed less as personal correction and more as communal preservation. As one participant explained, “It stopped being about looking a certain way. It became about staying alive.” Theological vocabulary shifted accordingly. Rather than centering discipline alone, clergy increasingly emphasized protection, responsibility, and safeguarding elders. Obesity was repositioned within a collective moral frame rather than an individualized behavioral one.

### Embodied Witness and Ministerial Exposure

Participants did not restrict discussion of obesity to congregants. In most interviews, reflection turned inward. Clergy described the pastoral role itself as physically demanding and often unsustainable. Long hours, food-centered fellowship, emotional labor, and irregular routines were normalized features of ministry. Several referenced colleagues who had died prematurely from hypertension, diabetes, or cardiovascular disease.

One pastor remarked, “We preach self-control on Sunday and eat at 10 p.m. after a meeting. That’s the culture.” Another stated, “This job will kill you if you let it.”

COVID-19 intensified awareness of these embodied risks. Clergy with personal diagnoses described heightened vulnerability. One participant shared, “I wasn’t taking care of myself. I ended up on dialysis for 9 years. That changes how you preach about the body.”

Participants consistently described forms of embodied leadership as a recurring theme across interviews. They emphasized that congregants observed their habits closely, and that everyday practices carried interpretive significance. As one participant noted, “They’re watching. If I don’t live it, I can’t teach it.”

Importantly, embodied witness was not framed as perfection. Several clergy described ongoing struggle and positioned transparency as formative rather than disqualifying. “I don’t stand up there pretending. I tell them where I’m working on myself.”

In these narratives, the pastoral body functioned simultaneously as instructional, symbolic, and relational. Health leadership was described as emerging not solely from expertise but from visibility, credibility, and lived example.

### Crisis as Moral Compression

Participants consistently described the pandemic as compressing time, consequence, and decision making. Funerals conducted under distancing restrictions, hospital visits by phone, and repeated exposure to congregational grief created what several clergy referred to as a “wake-up season.” Chronic disease was no longer experienced as a gradual decline, but as an immediate amplification of risk.

One clergy member stated, “You bury enough people and you start asking different questions.” Another reflected, “The process of change happened faster than we were ready for.”

Health initiatives that had previously been postponed were often launched during or shortly after peak mortality periods. Clergy described starting walking groups, virtual exercise challenges, sermon series focused on wellness, and partnerships with healthcare professionals.

These efforts were not described as the result of long-term strategic planning, but as moral responses to what they were witnessing. As one participant shared, “I couldn’t keep preaching hope and ignore what was killing us.”

Participants also described a shift in how they approached discomfort. Addressing obesity had previously felt socially sensitive, but after witnessing disproportionate mortality among those with metabolic conditions, hesitation decreased. As one participant noted, “It wasn’t comfortable. But it was necessary.”

Participant accounts in this domain reflect a form of interpretive acceleration, where mortality made risk immediate and visible. What had once been understood as long-term risk became a present and urgent concern, reshaping how clergy understood responsibility and action.

### Translational Authority Under Conditions of Mistrust

Across interviews, clergy described functioning as intermediaries between public health institutions and congregational communities. Historical mistrust surfaced repeatedly in participant narratives. Clergy referenced skepticism about vaccination, testing, and hospital systems. They acknowledged the legacy of medical exploitation and ongoing disparities that shaped congregational hesitation.

One pastor explained, “If I don’t explain it in a way that connects to their faith, they won’t receive it.” Another stated, “They trust me before they trust a press conference.”

Participants described interpreting epidemiological data, contextualizing vaccination within theological narratives, and responding to misinformation circulating through social media. Translation was described as requiring theological coherence rather than simple information transfer.

Several clergy reported integrating health guidance into sermons, prayer calls, and Bible studies. Biomedical language was reframed through scriptural and pastoral vocabulary without minimizing scientific content. “You have to put it in a language that honors both the science and the faith.”

This translational work extended beyond vaccination to obesity and chronic disease. Rather than presenting weight as a medical statistic alone, clergy situated it within narratives of stewardship, legacy, and communal continuity. Self-confidence in this mediating role was grounded less in technical training and more in vocational responsibility. “I’m not a doctor. But I’m responsible for the whole person.”

Public health engagement was consistently described as an expansion of pastoral care rather than a departure from it. Authority operated relationally. Trust, not credentialing, mediated uptake.

### Cross-Domain Integration

These domains operated simultaneously rather than sequentially. Participants described how the reframing of obesity as an existential threat deepened their reflection on embodiment, which in turn heightened moral urgency. This sense of urgency was linked to expanded forms of leadership, including how they communicated and translated health information within their congregations. In this way, shifts in one area reinforced changes in others, shaping how vulnerability and responsibility were understood.

Across interviews, participants’ accounts reflected elements consistent with behavioral constructs such as decisional balance and self-efficacy, though these were embedded within theological and relational meaning systems. Benefits of change were described in terms of survival, faithfulness, and protection of the congregation, while confidence was grounded in calling and accountability rather than individual autonomy. Processes of change were also framed collectively, with an emphasis on shared responsibility rather than individual action alone.

Clergy did not describe themselves primarily as implementers of weight loss programs. Instead, they spoke about undergoing shifts in how they understood their role, with chronic disease prevention becoming more closely tied to pastoral identity. COVID-19 did not introduce health into ministry, but brought it into sharper focus. Obesity was no longer a peripheral concern, but a visible marker of vulnerability. The body moved from metaphor to something that required ongoing attention, and pastoral leadership expanded to include interpreting and responding to public health concerns within the context of structural inequity.

## Discussion

### Syndemic Disruption and Theological Reordering

This study examined how African American clergy interpreted and responded to the convergence of COVID-19 and chronic disease vulnerability, with particular attention to how these conditions reshaped theological meaning, pastoral identity, and health-related leadership. Findings show that COVID-19 functioned not simply as a public health crisis but as a syndemic disruption that reordered pastoral priorities within Black congregations. Syndemic theory highlights the clustering and interaction of disease within contexts of social inequality (Singer et al., 2017).

Rather than unfolding independently, COVID-19 intensified preexisting chronic disease disparities through both biological and structural pathways. As Horton (2020) argues, the pandemic is best understood as syndemic because its severity is shaped by the social conditions that render certain populations disproportionately vulnerable.

What this study adds beyond prior faith-based health research is a focus on how syndemic exposure is interpreted within theological systems and incorporated into pastoral identity, rather than examining clergy primarily as facilitators of discrete health interventions. For clergy in this sample, obesity and metabolic illness became visible as amplifiers of risk embedded in racialized structural environments. Participants did not describe COVID-19 as random misfortune. They interpreted disproportionate mortality as the embodied consequence of historical and contemporary inequities, including residential segregation, environmental exposure, constrained food access, and healthcare exclusion (Bailey et al., 2019; Nardone et al., 2020). This shift represents more than structural awareness. It reflects a theological reframing of chronic disease from individual lapse to structurally mediated vulnerability.

The pandemic functioned as what some scholars describe as a moral shock, an event that disrupts normative assumptions and compels ethical reevaluation (Jasper, 2018). Clergy described changes in how the body was preached, discussed, and modeled. The metaphor of the body as temple was no longer rhetorical reinforcement. It carried greater urgency. Stewardship language intensified, not as moralistic discipline, but as a form of communal preservation under conditions of visible loss.

This theological reordering expands prior scholarship on faith-based health promotion, which has often centered intervention efficacy rather than crisis-induced meaning transformation. The findings suggest that syndemic conditions do more than heighten disease burden. They can prompt doctrinal reinterpretation and shifts in institutional priorities. Chronic disease prevention becomes not simply supportive programming but an integrated pastoral mandate shaped by structural awareness and embodied loss.

### Structural Racism and Embodied Vulnerability

Participants consistently articulated that chronic disease disparities within their congregations were not reducible to individual choice. Their narratives aligned with public health scholarship identifying structural racism as a fundamental cause of health inequity (Williams et al., 2019). Structural conditions shape food environments, neighborhood safety, stress exposure, employment precarity, environmental toxicity, and access to preventive care. These forces accumulate over time, influencing obesity prevalence and metabolic disease trajectories in patterned and predictable ways.

Although clergy did not invoke academic terminology such as “fundamental cause theory,” they described its lived manifestation. They spoke of congregants navigating limited grocery options, working multiple jobs with irregular hours, residing in neighborhoods with constrained recreational space, and encountering barriers to consistent medical care. In these accounts, obesity was not framed as isolated failure. It was situated within constrained structural possibility.

This interpretive stance represents a significant theological movement. Rather than locating chronic disease primarily within individualized moral discipline, clergy articulated a framework of communal accountability and shared burden. Embodiment was understood as shaped by history and policy as much as by appetite or willpower. This perspective resonates with scholarship on embodiment demonstrating that social inequality becomes biologically inscribed across the life course (Krieger, 2011). Congregational bodies were recognized as bearing the imprint of structural disadvantage.

Importantly, this reframing alters the moral terrain of obesity discourse. Research on weight stigma demonstrates that narratives of personal blame undermine engagement and intensify psychological distress (Puhl & Suh, 2015). By situating obesity within structural and communal vulnerability, clergy narratives shifted the focus from correction to protection. Chronic disease became a collective condition requiring shared response rather than a marker of individual moral lapse.

Under syndemic conditions, structural racism was not an abstract analytic category. It became visible through disproportionate hospitalization and loss. Clergy functioned as interpreters of this embodied inequity, translating structural constraint into theological language without dissolving its material reality. In doing so, they bridged public health analysis and pastoral care, expanding the scope of religious leadership to include structural consciousness alongside spiritual guidance.

### Moral Mediation of Behavioral Constructs

Although behavioral constructs were evident across narratives, they were consistently mediated through theological anthropology. Decisions about obesity, chronic disease engagement, and public health leadership were not framed as neutral cost–benefit calculations. Decisional balance functioned as moral discernment. Benefits were articulated in terms of faithfulness to calling, longevity in ministry, protection of elders, and preservation of congregational life. Costs were framed not as inconvenience alone but as neglect of pastoral responsibility or diminished witness.

This finding complicates purely cognitive models of behavior change. In faith contexts, behavior is not simply the outcome of perceived susceptibility or perceived benefit. It is interpreted within moral and doctrinal frameworks. Religion operates not only as social support but as a comprehensive meaning system that shapes identity, obligation, and communal norms (Park, 2013). As Idler (2014) argues, religion influences health through moral directives, embodied practices, ritual reinforcement, and collective expectation. The present findings demonstrate this mediation dynamically under crisis conditions. Behavioral constructs were present, but they were refracted through theological interpretation.

Self-efficacy similarly acquired theological grounding. Clergy did not describe agency solely in terms of personal mastery or skill acquisition. Rather, they framed action as alignment with divine purpose and accountability to community. Confidence emerged from calling as much as competence. In this sense, Bandura’s construct of self-efficacy operates differently within relational theology, where autonomy is secondary to obedience, stewardship, and collective responsibility.

Under syndemic pressure, this moral mediation intensified. When obesity and metabolic illness became linked to visible mortality, behavioral decisions carried heightened spiritual significance. Readiness to act was not simply a function of information exposure. It was catalyzed by moral urgency embedded in theological identity.

For religion and health scholarship, these findings suggest that behavioral theory applied in faith settings must account for spiritual mediation. Constructs such as readiness, decisional balance, and self-efficacy are not invalidated in religious contexts, but they are transformed. They operate within frameworks of vocation, covenant, and communal accountability. Integrating theological anthropology into behavior change models may therefore enhance both analytic precision and intervention design in faith-based health promotion.

### Embodied Authority and Relational Trust

Embodied leadership was a central theme in how participants described their role. Clergy described their bodies not merely as private vessels but as visible texts interpreted by congregants. Eating patterns, weight changes, visible fatigue, and self-care practices were understood as public signals of spiritual coherence. In this sense, pastoral embodiment functioned as moral evidence, with authority enacted through visible alignment between preaching and practice.

Within historically marginalized communities marked by justified medical mistrust, relational authority carries heightened significance (Hammond et al., 2021). Trust in biomedical institutions is shaped by legacies of exploitation and ongoing inequity. In this context, clergy operate within an existing relational infrastructure that predates the crisis. Research demonstrates that trust is a critical determinant of health message uptake in African American communities (Cuevas et al., 2016). Participants described drawing on this relational capital to interpret public health guidance, contextualize risk, and address skepticism circulating through digital networks.

Clergy influence was not described as merely instrumental or informational, but as interpretive. Participants explained how they embedded vaccination, masking, and chronic disease awareness within theological narratives and congregational story. Health recommendations were framed through scriptural language, communal responsibility, and pastoral care. This aligns with scholarship describing the Black church as a moral community that shapes norms through shared ritual, testimony, and collective memory (Lincoln & Mamiya, 1990). Meaning was co-constructed rather than simply transmitted.

Embodied authority operated at the intersection of visibility, trust, and narrative framing. Pastoral bodies became sites where theological commitment, health practice, and communal accountability converged. Under syndemic conditions, this convergence intensified.

For public health systems, overlooking this interpretive authority risks strategic misalignment. Clergy is not simply dissemination endpoints within communication chains. They function as co-constructors of health meaning. Engagement strategies that treat faith leaders as passive conduits underestimate the relational and theological mediation through which health behaviors are understood and negotiated within Black congregational life.

### Cultural Continuity and Adaptive Innovation

Food-centered fellowship represented both cultural continuity and structural tension within congregational life. Shared meals function as more than dietary practices. They reinforce hospitality, mutual care, celebration, and intergenerational belonging. Obesity-related risk therefore intersects not only with individual behavior but with deeply embedded ritual and communal identity.

Rather than abandoning these traditions, clergy described engaging in adaptive reinterpretation. This dynamic reflects what sociologists describe as institutional bricolage, the capacity of communities to modify inherited practices while preserving symbolic coherence (Cleaver, 2012). Participants reframed portion norms, introduced healthier food options without eliminating traditional dishes, encouraged collective challenges, and integrated health language into existing celebratory rhythms. Importantly, change was layered onto tradition rather than imposed in opposition to it.

This strategy carries analytic significance. Health promotion efforts that position cultural practices as obstacles often generate resistance or moral fatigue. In contrast, adaptive innovation preserves symbolic continuity while recalibrating risk exposure. Scholarship on culturally grounded intervention design demonstrates that alignment with identity enhances uptake and sustainability (Hammond et al., 2021). The clergy in this study intuitively enacted this principle by embedding metabolic health discourse within fellowship rather than displacing it.

The pandemic intensified awareness that tradition without adaptation could inadvertently amplify vulnerability. Mortality made visible the costs of unexamined continuity. Yet participants did not describe cultural abandonment. They described negotiation. Crisis accelerated reinterpretation without eroding communal identity.

This finding suggests that within Black church contexts, obesity prevention may be most durable when it honors food as relational practice while gradually reshaping its health implications. Cultural continuity and health promotion are not mutually exclusive. Under syndemic pressure, they become sites of creative theological and institutional recalibration.

### Crisis as Accelerator of Institutional Identity

A central contribution of this study is the identification of crisis as an accelerator of institutional identity rather than its origin. Faith communities in this sample were not newly introduced to health engagement during COVID-19. Health fairs, wellness ministries, and chronic disease discussions existed prior to the pandemic. What shifted was not participation alone but integration. Chronic disease prevention moved from auxiliary programming to a defining feature of pastoral vocation.

Religious coping theory suggests that crisis can catalyze meaning reconstruction and reinforce collective identity under threat (Pargament, 2011). Participants described grief and repeated exposure to mortality as clarifying moments that reshaped vocational self-understanding. The pandemic compressed abstract epidemiological risk into visible congregational loss. This compression altered urgency, moral framing, and perceived responsibility. Health advocacy became less discretionary and more intrinsic to shepherding.

From an institutional perspective, this shift reflects movement from episodic mobilization to identity-level incorporation. Rather than treating health promotion as a responsive initiative triggered by emergency conditions, clergy articulated it as an enduring dimension of pastoral calling. This distinction is analytically important because temporary mobilization reflects adaptive response, whereas identity integration reflects structural transformation.

These findings suggest that health crises may function as inflection points in institutional self-definition within religious communities. When structural vulnerability becomes embodied through loss, theological interpretation may reorganize ministry priorities in durable ways. Future research should examine whether such crisis-induced acceleration persists beyond acute phases and whether similar identity shifts occur across denominational, regional, or interfaith contexts.

### Implications for Religion and Health Scholarship

This study advances religion and health scholarship by positioning clergy leadership within a syndemic framework rather than limiting analysis to individual chronic disease interventions. By examining how COVID-19 intensified the interaction between structural inequality and metabolic vulnerability, the findings move beyond program evaluation to show how crisis conditions can reshape theological interpretation and expand pastoral roles.

A central contribution of this study is demonstrating that behavioral constructs such as decisional balance and self-efficacy do not operate in isolation within faith settings. Instead, they are shaped by theological reasoning, moral responsibility, and communal identity. This challenges approaches that treat behavior change as purely cognitive and highlights the importance of incorporating theological anthropology into religion and health research. In this context, behavior is tied not only to individual decision-making, but also to meaning, calling, and collective accountability.

The findings also contribute to scholarship on structural racism and religion by showing how clergy interpret chronic disease disparities as the result of systemic constraints rather than individual failure. This shift reframes obesity from a stigmatized personal issue to a shared marker of embodied inequity, with important implications for how health disparities are understood and addressed in faith-based settings.

Although religion and health research increasingly recognizes the role of faith institutions in addressing health inequities (Koenig, 2022), fewer studies examine how theological meaning systems change under conditions of acute structural stress. By focusing on interpretive shifts rather than intervention outcomes alone, this study broadens the scope of the field and suggests that crisis moments can serve as turning points in how clergy understand health as part of their leadership.

The findings also point to how these shifts in meaning and identity may carry forward into ongoing health efforts within congregations. When chronic disease prevention becomes part of how clergy understand their role, it is more likely to appear consistently in sermons, ministry activities, and everyday pastoral care rather than as a short-term response to crisis. Several clergy described starting or expanding efforts such as wellness challenges, partnerships with healthcare professionals, and the regular incorporation of health messages into worship and counseling. These practices provide a foundation for continuity beyond the height of the pandemic. Over time, this type of integration may support increased participation in health programs, stronger health literacy, and greater engagement in preventive behaviors such as physical activity, nutrition changes, and routine screening. While this study does not measure these outcomes directly, the findings suggest that shifts in meaning and pastoral identity can help sustain health-related efforts within faith communities.

Future research should examine how syndemic disruption influences doctrinal emphasis, congregational norms, and institutional identity across different settings. While this study focuses on African American clergy in Houston, the insights may extend to other contexts where faith leaders help communities make sense of health and inequality. Similar processes may be present in immigrant congregations, rural churches, and other marginalized communities. Although the language and institutional structures may differ, the broader pattern of crisis-driven reinterpretation and evolving leadership roles may be relevant across settings.

### Implications for Practice and Policy

These findings reinforce the need for sustained, reciprocal partnership between public health systems and African American faith institutions. Clergy possess relational authority that can enhance trust and message uptake, particularly in communities shaped by historical and contemporary medical injustice (Berkley-Patton et al., 2022). Public health engagement strategies should therefore move beyond episodic outreach during crisis periods toward long-term institutional collaboration.

Partnership must be developmental rather than transactional. Rather than approaching churches solely as dissemination sites, health agencies should support capacity-building initiatives that equip clergy with sustained access to evidence-based health education resources, implementation support, and technical consultation. Funding mechanisms at local and state levels could prioritize faith–public health collaborative grants that include clergy leadership as co-investigators or advisory partners rather than peripheral endorsers.

Seminary curricula and denominational leadership development structures represent underutilized intervention points. Integrating population health literacy, chronic disease prevention, stress management, and structural determinants of health into pastoral formation may reduce reliance on crisis-driven mobilization and support proactive health leadership. Such integration situates embodiment within theological training rather than as a reactive add-on.

Public health agencies designing obesity and chronic disease interventions in Black communities should incorporate clergy during formative planning phases. Co-design processes that allow theological framing, cultural continuity, and congregational rhythms to shape intervention structure are more likely to achieve sustainability than externally imposed models.

At the policy level, strategies addressing chronic disease disparities must recognize that structural determinants and spiritual institutions operate within the same social ecology. Effective health equity frameworks should include faith institutions as components of community infrastructure, alongside schools, clinics, and housing systems. Ignoring either structural determinants or the moral institutions that mediate communal meaning constrains long-term impact.

### Limitations

This study reflects the perspectives of 10 clergy serving predominantly African American congregations within a single metropolitan region in the Southern USA. While Houston provides a context marked by significant racial health disparities and a robust Black church infrastructure, regional political climates, denominational cultures, and public health environments may shape clergy interpretation differently in other settings. The small, purposive sample across multiple Protestant denominations provides context-specific, exploratory insight and does not support statistical generalizability or broad transferability across diverse ecclesial contexts.

Although participants represented variation in pastoral role, including senior and associate clergy, the sample was predominantly male, and women clergy may experience embodied leadership and health discourse differently. In addition, demographic variables such as age, education level, income, and specific denominational affiliation were not systematically collected, limiting the ability to assess within-group variation and potential sources of bias.

The timing of data collection may also have influenced narrative intensity. Interviews were conducted during a period still proximate to acute pandemic conditions, and clergy reflections may reflect heightened awareness of mortality and vulnerability. Interpretations may evolve as greater temporal distance from the pandemic develops.

As a qualitative, interpretive phenomenological inquiry, the study prioritizes meaning-making over measurable health outcomes and does not assess behavioral change, congregational biometric indicators, or sustained program implementation. Researcher positionality within public health and faith-based health initiatives may have shaped analytic sensitivity, despite the use of reflexive safeguards.

Future longitudinal and mixed-methods research is needed to examine whether theological reframing translates into sustained institutional change, measurable health outcomes, and durable shifts in congregational norms across diverse settings.

## Conclusion

The COVID-19 pandemic brought into sharper focus what had long been structurally embedded: the convergence of chronic disease, racialized inequity, and infectious threat within African American communities. This study examined how African American clergy interpreted and responded to the convergence of COVID-19 and chronic disease vulnerability, and how these conditions reshaped theological meaning, pastoral identity, and health-related leadership within Black church contexts. For the clergy in this study, this was not experienced as abstract epidemiology, but through funerals conducted under restriction, hospitalizations among congregants with metabolic illness, and the steady weight of communal grief. In this context, obesity and related chronic conditions were no longer understood primarily as matters of personal discipline, but as visible expressions of structural vulnerability.

COVID-19 functioned as a syndemic disruption that accelerated theological and institutional change. Health engagement did not begin with the pandemic, but became more fully integrated into pastoral identity. Chronic disease prevention shifted from auxiliary programming to a core part of shepherding. The body was no longer invoked only as metaphor, but as something requiring ongoing care, responsibility, and attention within a racialized health landscape.

Behavioral constructs such as readiness, decisional balance, and self-efficacy were present, but operated within moral and theological frameworks rather than purely cognitive ones. Decisions about health were framed in terms of discernment, vocation, and protection of the congregation. Agency was grounded in calling and relational responsibility. Change unfolded not only through access to information, but through a sense of urgency shaped by loss and lived experience.

Clergy functioned as interpretive leaders at the intersection of public health and congregational life. Their authority was rooted less in formal medical expertise and more in relational trust built over time. In communities shaped by structural racism and justified medical mistrust, this trust played a central role in how health information was received and acted upon. Health messages carried weight not only because they were evidence-based, but because they were translated into theological language and situated within shared experience.

These findings point to the need for approaches to chronic disease disparities that engage both structural conditions and the institutions that shape meaning, identity, and collective action. Within many Black communities, the church continues to serve as a central site of this work. Under syndemic conditions, pastoral identity can expand to include health advocacy as part of core ministry rather than as an added responsibility.

The pandemic made visible the close connection between spiritual leadership and public health. Recognizing clergy as active interpreters and shapers of health meaning, rather than peripheral partners, is critical for developing more effective and sustainable strategies to advance health equity in historically marginalized communities.
